# Back Pain and Right Leg Swelling: Unusual Presentations of May-Thurner Syndrome

**DOI:** 10.7759/cureus.35984

**Published:** 2023-03-10

**Authors:** Eleanor Dunlap, Suzanna Fitzpatrick, Khanjan Nagarsheth

**Affiliations:** 1 Vascular Surgery, University of Maryland Medical Center, Baltimore, USA; 2 Vascular Surgery, University of Maryland School of Medicine, Baltimore, USA

**Keywords:** may thurner syndrome (mts), may-thurner's syndrome, pain, vascular compression, iliac vein stenosis, venous compression

## Abstract

May-Thurner (MT) syndrome refers to compression of the left common iliac vein by the right common iliac artery. Symptoms reported are generally left-sided leg swelling or pain. It is unusual for patients to report right-sided symptoms that are alleviated by treating MT compression. This case series describes three patients who had right-sided symptoms caused by left-sided venous compression. A retrospective chart review identified three patients over a year who presented with a variety of symptoms, including right-leg pain and swelling, and underwent treatment with left-sided venous compressions with a resolution of symptoms. Three patients were identified with right-sided back and flank pain. Venography with intravascular ultrasound (IVUS) showed the MT compression was greater than 75% in each case (mean 80.3% with a range of 75.7%-95%), and all patients were treated by decompressing the venous outflow obstruction by stenting the left common iliac vein, which relieved their symptoms. Venous compressions that occur on the anatomical left side can lead to right-sided symptoms. In patients reporting right-sided back and flank pain, MT should be considered in the differential diagnosis.

## Introduction

May-Thurner (MT) syndrome was first described in a cohort of patients with left-sided deep vein thromboses (DVT) as a vascular anatomic variant caused by compression of the left common iliac vein by the right common iliac artery [1]. This syndrome is often detected after a person develops symptoms of venous stasis, including left leg swelling, heaviness, pain, venous stasis ulcers, and paresthesias [2]. Not surprisingly, venous stasis caused by MT results in an increased propensity for DVT. Some studies have even described that the higher frequency of left-sided than right-sided DVT may be explained by the prevalence of MT in the population [3]. The symptoms of MT syndrome are mostly reported on the left side due to a direct reduction in the venous outflow from the vessels of the left leg. The few reports of right-sided symptoms secondary to MT usually reference specific variations in vessel anatomy or enlargement of pelvic structures leading to direct compression of right-sided leg veins [4-5].

Herein, we present a case series of three patients who presented with right-sided symptoms that were caused by left-sided iliac vein compressions.

## Case presentation

Methods

A retrospective chart review identified three patients over a one-year period who presented with a variety of symptoms, including right-leg pain and swelling. These patients underwent treatment with left-sided venous compressions, resulting in the resolution of these right-sided symptoms. These charts were reviewed for patient demographic data, symptoms, percent of compression, and treatment. Prior to the start of this project, an Institutional Review Board (HP-00085462) was obtained. Each patient gave informed consent for their de-identified images and clinical information to be used, and careful attention was given to the protection of human rights.

Results

The average age of the patients was 46.5 years (range 34-67 years), and all patients were women. All of the patients had right-sided back and flank pain. Two of the patients had right-leg pain or swelling. One patient reported headaches. In all cases, the percent of venous compression was greater than 75% (mean 80.3% with a range of 75.7%-95%), and all patients were treated by decompressing the venous outflow obstruction by stenting the left iliac vein (Table 1).

**Table 1:** History of symptoms and treatment IVUS: intravascular ultrasound

Patient	Age	History	Symptoms	Percent compression on IVUS	Treatment
1	67	Stroke, hypertension, gastrointestinal reflux	Pain and swelling in the right and left leg	95%	Left common iliac venous stent placement with a 14 mm x 140 mm Cook vena stent
2	62	Hypertension, obesity	Right-sided back pain, pain and swelling in the right leg	76%	Left common iliac venous stent placement with a 14 mm x 140 mm Cook vena stent
3	41	Ehlers-Danlos syndrome, fibromuscular dysplasia, gastroparesis, carotid stenosis	Right-sided back pain, swelling in the left leg, and headache	77.7%	Left common iliac venous stent placement with a 14 mm x 140 mm Cook vena stent

**Figure 1:**
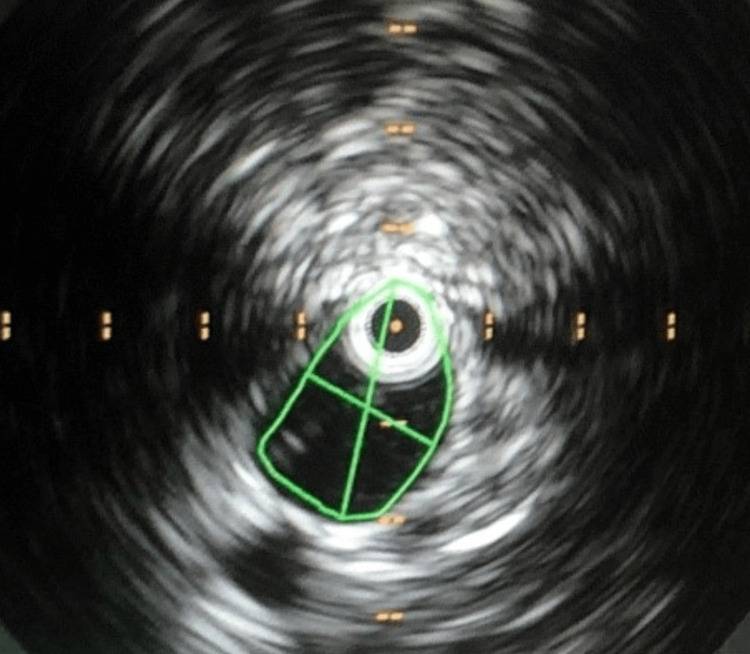
IVUS image of left iliac vein compression

IVUS imaging was used to measure the left iliac vein compression prior to stenting (Figure 1).

**Figure 2:**
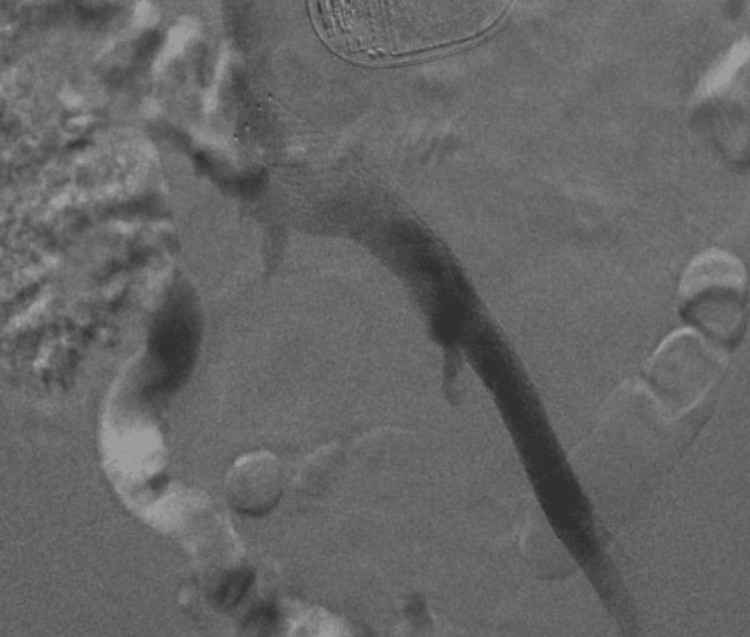
Venogram after iliac vein stenting

After stent placement, a repeat venogram was performed to confirm placement and resolution of venous outflow obstruction (Figure 2).

## Discussion

MT syndrome is often diagnosed once venous congestion reaches the level of chronic changes to the tissues or a thrombus develops. Khan et al. describe that MT affects more women than men between the ages of 20 and 50 years. When symptoms are unilateral leg swelling, the differential diagnosis includes, but is not limited to, deep vein thrombosis (DVT), cellulitis, or venous insufficiency [6]. Further imaging is needed to identify venous compression.

Although the exact prevalence of MT is unknown, a recent study found that 31% of patients with iliofemoral DVT had MT [7]. Kibbe et al reported that up to two-thirds of asymptomatic patients had left common iliac vein compression greater than 25% and almost one-fourth had over 50% compression, which indicates that MT anatomy occurs more often than it is reported [8].

The diagnosis of MT syndrome is based on clinician suspicion and diagnostic testing. Computed tomography (CT), magnetic resonance imaging (MRI), or duplex ultrasound can evaluate the central venous system and note if there is extrinsic compression of the vein; however, venography with IVUS is the current gold standard for visualizing the degree of compression of the iliac vein and establishing a diagnosis of MT [9].

In these cases, venous duplex ultrasound was performed first due to leg swelling and pain. Venography with IVUS was performed to assess for right iliac vein compression. Right iliac vein compression, while rare, has been reported in the literature [10]. When no right iliac compression was found, attention was focused on the left iliac vein, and there was compression noted. There are no other reports of left-sided iliac vein compression causing right-sided symptoms in the literature. Treating the compression on the left resolved these patients’ pain and swelling on the right, in addition to one patient’s chronic headaches.

## Conclusions

Venous compressions that occur on the anatomical left side can lead to right-sided symptoms. Advancements in modern diagnostic imaging capabilities through CT scans have led to an increase in the diagnosis of venous compressions. In each of these cases, the treatment of left-sided venous compression alleviated the right-sided symptoms, decreasing pain and swelling, and highlighting the need to consider contralateral venous compressions in differential diagnoses.
